# Understanding gastro-oesophageal reflux disease: a patient-cluster analysis

**DOI:** 10.1111/j.1742-1241.2008.01929.x

**Published:** 2008-12

**Authors:** A King, C MacDonald, C Örn

**Affiliations:** 1 CLEARRichmond, Surrey, UK; 2 AstraZeneca R&DMölndal, Sweden

## Abstract

**Objective:**

To determine whether patients with gastro-oesophageal reflux disease (GERD) can be grouped according to the physical and psychological impact of their disease.

**Methods:**

In this multinational study, 7713 primary care physicians (PCPs) and gastrointestinal (GI) specialists took part in a structured online survey to determine how they perceive the clinical and psychological needs of their GERD patients, based on their three most recent consultations. Patients were grouped according to one of the five clusters that were subjectively developed based on preceding qualitative research.

**Results:**

Findings are reported for 1157 respondents (875 PCPs, 282 GI specialists), who reviewed 3471 patient records. Two of the five original clusters were collapsed because of overlapping characteristics, giving rise to three patient clusters. Patients with ‘long-term, disrupting GERD’ (39%) had symptoms considered to have not only high physical but also psychological impact. Patients with ‘recurrent, distressing GERD’ (14%) experienced both physical and psychological impact and were worried about the recurrent, restrictive nature of their disease or the possibility of having a more serious underlying condition. Patients with ‘inconveniencing GERD’ (48%) had less frequent symptoms with overall lower impact. Overall, there was a trend for GI specialists to more likely see patients at higher clinical need than PCPs.

**Conclusions:**

Patients with GERD can generally be classified according to the physical and psychological impact of their disease. Recognition that such patients have different needs may facilitate improved management of GERD by allowing treatment to be tailored according to the patient's need.

**What's known**

- Physicians generally appreciate that patients diagnosed with gastro-oesophageal reflux disease (GERD) are a heterogeneous population with therapeutic needs that differ according to the impact of their disease.
- However, the management of GERD remains far from optimal, as highlighted by prevalent self-medication with over-the-counter agents even among patients receiving prescription therapy.

**What's new**

- The findings of this survey of primary care physicians and gastrointestinal specialists indicate that patients with GERD can generally be grouped according to the perceived physical and psychological impact of their disease.
- Recognition that such patients have different needs, through understanding of the three population clusters identified in this study, may facilitate improved management of GERD by allowing treatment to be tailored according to the patient's need.

## Introduction

Gastro-oesophageal reflux disease (GERD) is a highly prevalent condition that is associated with a diverse range of symptoms, of which heartburn and regurgitation are most common (1,2). GERD is routinely encountered by physicians in daily practice (3).

Physicians generally appreciate that diagnosed GERD patients are a heterogeneous population with different needs. Indeed, the symptoms of GERD may be either relatively mild or infrequent in some patients, while in others they can be sufficiently bothersome to disrupt the individual's physical, social and emotional well-being, necessitating aggressive treatment (4,5). As such, a variety of therapeutic options are available for the treatment of GERD, ranging from lifestyle modifications and over-the-counter (OTC) agents to prescription acid-suppressive therapy (6).

Optimal management of GERD in clinical practice aims to alleviate symptoms, heal erosions, prevent long-term complications (in at-risk patients) and improve patient well-being. Acid-suppressive therapy with proton pump inhibitors (PPIs) is generally regarded as the treatment of choice in this regard because of well-documented efficacy and favourable tolerability (6). However, self-medication with OTC agents such as antacids is common, even among patients receiving prescription therapy. A recent survey of GERD patients, for example, found that 20.5% were taking some form of OTC medication in addition to their prescription therapy (7). This may be explained by the fact that the symptoms and impact on quality of life associated with GERD often persists despite treatment (1,7,8). Reliance on OTC medication may also be related to the widespread perception among GERD sufferers that it is a trivial disease without long-term health consequences (9). Taken together, these findings outline the need for improved management of GERD in clinical practice. This study aimed to determine whether cluster analysis of patients with GERD, in terms of physician-perceived clinical and emotional needs, would create a foundation for improved patient management by allowing treatment to be tailored according to the patient's need.

## Methods

A survey was conducted to gather data on how physicians perceive the clinical and emotional needs of their GERD patients. Physicians [primary care physicians (PCPs) and gastrointestinal (GI) specialists] in France, Germany, Italy, the UK and the USA took part in the research, which was preceded by an initial qualitative phase during which in-depth interviews were conducted to establish how physicians treat GERD and their perceptions of the treatment goals and priorities for different patients (unpublished observations). On the basis of this research, five patient clusters were subjectively described:

1. Patients with occasional, but inconvenient symptoms of GERD where the patient demands an instant solution.
2. Patients with intermittent, bothersome symptoms of GERD that are likely to be attributed to lifestyle issues.
3. Patients with recurrent, persistent symptoms of GERD, for whom the current medication provides inadequate relief of symptoms and where the patient may be worried about their condition.
4. Patients with symptoms of GERD that have persisted for a long time; symptoms may be controlled by medication but return if PPIs are discontinued and there is a concern about long-term complications.
5. Patients with disruptive symptoms of GERD who have current evidence of disease.

These five clusters were used to develop pen portraits that were used in this quantitative research, in which a secured web-link was sent to 7713 PCPs and GI specialists in the participating countries (quotas having been set to ensure a representative sample of physicians in each country). A small financial incentive, commensurate with the length of the questionnaire, was offered as part of completing the survey, and reminders were sent once the web-link had been conveyed to potential participants to speed up recruitment and completion of the survey. Physicians were first asked to complete a number of screening questions, to determine their suitability for inclusion in the study. Only those physicians whose year of qualification in their particular specialty were between 1976 and 2002, and were actively treating patients with acid-related disorders, were allowed to participate. Further screening criteria were based on PPI prescription volume; PCPs were required to have written at least 10 PPI prescriptions for acid-related disorders in the last 4 weeks, while GI specialists must have written at least 20 PPI prescriptions in the same time period.

As part of the online survey, eligible physicians were subsequently asked to recall the last three patients with GERD whom they had treated. For each patient, physicians were asked to evaluate statements concerning each patient's physical and psychological impact of GERD and their attitudes to their disease, using six-point Likert scales (Table 1), and thereafter to assign him/her to one of the five clusters outlined above. The physical impact of GERD was determined subjectively based on responses to statements evaluating symptom frequency, severity, risk of future erosions/complications, level of symptom control, history of GERD and physical evidence of the disease. The psychological disease impact was similarly determined based on responses to statements on the relationship of symptoms to the patient, levels of anxiety about symptoms, patient distress, symptom-related disruption, patients’ interest in learning about their condition and likelihood of compliance with physician recommendations. All data were analysed descriptively.

**Table 1:** Characteristics for evaluation of the physical and psychological impact of GERD and patients’ attitudes to their disease

Frequency of symptoms (1 = frequent; 6 = occasional)
Severity of symptoms (1 = severe; 6 = mild)
Relationship of symptoms to the patient (1 = symptoms are linked to patient behaviour; 6 = symptoms are disease related)
Risk of future erosions or complications (1 = high; 6 = low)
Level of symptom control (1 = not controlled; 6 = controlled)
History of GERD (1 = long-time sufferer; 6 = only started to suffer from symptoms recently)
Level of anxiety about symptoms (1 = anxious; 6 = not anxious)
Level of patient distress (1 = clearly distressed; 6 = not distressed)
Disruption associated with symptoms (1 = very disruptive; 6 = low)
Physical evidence of disease (1 = physical evidence; 6 = no current physical evidence)
Level of patient interest in learning about his/her condition (1 = none; 6 = active interest)
Patient's likelihood to comply with physician's recommendations (1 = not likely to comply; 6 = likely to comply)
Level of sleep disruption (1 = disrupted; 6 = not disrupted)

GERD, gastro-oesophageal reflux disease.

## Results

A total of 1157 physicians (875 PCPs, 282 GI specialists) participated in the study (15% response rate), and around 30% were from the USA (Table 2). In accordance with the screening criteria, the majority of PCPs (97%) had written at least 10 PPI prescriptions for acid-related disorders in the last 4 weeks, while 89% of GI specialists had written at least 20 PPI prescriptions in the same time period. The most commonly prescribed PPI for both physician groups was generic omeprazole.

**Table 2:** Characteristics of physicians who participated in the study

	Primary care physicians (*n* = 875)	Gastrointestinal specialists (*n* = 282)
**Country, *n* (%)**		
France	160 (18)	56 (20)
Germany	154 (18)	54 (19)
Italy	155 (18)	45 (16)
UK	177 (20)	60 (21)
USA	229 (26)	77 (27)
**Year of qualification, *n* (%)**		
1976 to 1985	358 (41)	73 (26)
1986 to 1995	345 (39)	108 (38)
1996 to 2002	172 (20)	101 (36)

**Figure 1:**
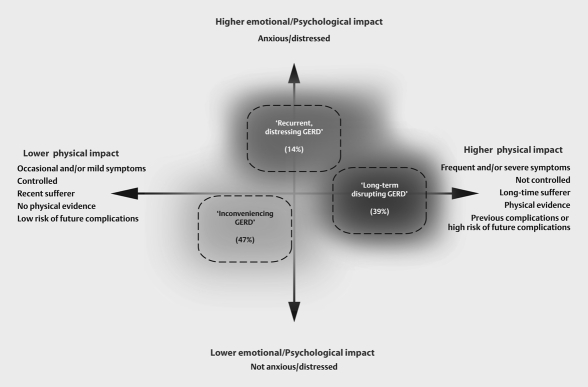
Stylistic interpretation of the two-dimensional spectrum of physical and psychological impact in patients with GERD, according to patient cluster

A total of 3471 patient records were reviewed, and physicians were able to classify all of their patients according to one of the five pen portraits. Clusters 1 and 2 showed overlapping characteristics, as did clusters 4 and 5. Based on the evaluation of physician-perceived impact of GERD on physical and psychological dimensions, therefore, the clusters were reduced to the following three (Figure 1):

- Patients with ‘long-term, disrupting GERD’.
- Patients with ‘recurrent, distressing GERD’.
- Patients with ‘inconveniencing GERD’.

**Figure 2:**
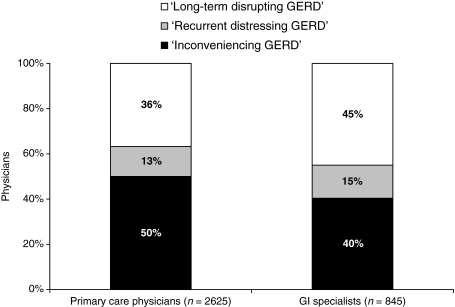
Distribution of patient clusters, according to physician profile

Overall, there was a trend for GI specialists to more likely see patients at highest clinical need (i.e. patients with high physical and psychological impact) than PCPs (Figure 2), and there were no relevant by-country differences.

### Patients with ‘long-term, disrupting GERD’

Patients in this cluster, which accounted for over one-third of the GERD patient population (39%; Europe, 40%; USA, 35%), had suffered GERD symptoms for a long time (59% of patients had experienced symptoms for > 2 years) and, compared with the other clusters, had more frequent or severe symptoms that were disrupting daily life (Table 3). Consequently, such symptoms were perceived by physicians to confer high physical impact as well as psychological impact. Overall, patients in this cluster either already had complications or had a high risk of future complications. Indeed, when asked to describe such patients in terms of ‘high-risk of future complications’, physicians felt that this statement described 36.5% of evaluable patients in this cluster (Table 3).

**Table 3:** Statement agreement (physicians’ perceptions)

	Cluster, % (*n*)		
	
	‘Inconveniencing GERD’ (*n* = 1653)	‘Recurrent, distressing GERD’ (*n* = 473)	‘Long-term, disrupting GERD’ (*n* = 1345)
Frequent symptoms	15.7 (260)	32.6 (154)	38.9 (523)
Severe symptoms	15.7 (259)	23.5 (111)	30.6 (411)
Symptoms are linked to patient behaviour	29.0 (479)	17.5 (83)	16.4 (221)
High risk of future complications	14.8 (244)	22.4 (106)	36.5 (491)
Symptoms are not controlled	18.3 (302)	26.6 (126)	26.0 (350)
Long-term sufferer of GERD symptoms	18.4 (304)	29.6 (140)	41.1 (553)
Patient is anxious about symptoms	20.1 (333)	31.5 (149)	30.5 (410)
Patient is clearly distressed	19.4 (320)	31.1 (147)	28.3 (381)
Symptoms are very disruptive	16.5 (272)	28.3 (134)	27.0 (363)
Physical evidence of disease	17.6 (291)	19.5 (92)	29.3 (394)
Patient has their sleep disrupted	17.3 (286)	22.6 (107)	25.9 (349)
Patient feels that his/her symptoms are largely attributable to lifestyle	6.8 (113)	4.0 (19)	3.9 (52)
Patient is very frustrated by their GERD and feels they can no longer cope	2.6 (43)	5.9 (28)	4.8 (64)
Patient feels unhappy because the disease restricts his/her life	3.9 (64)	8.9 (42)	6.9 (93)
Patient sometimes worries that there might be something more serious underlying their symptoms	5.1 (85)	13.5 (64)	12.9 (174)
Patient demands an ‘instant’ solution	16.9 (280)	9.7 (46)	12.3 (165)

GERD, gastro-oesophageal reflux disease.

### Patients with ‘recurrent, distressing GERD’

Patients in this cluster accounted for the smallest portion of the GERD population (14%; Europe, 13%; USA, 16%). Overall, physicians felt that such patients were distressed by their GERD symptoms, having physical but also psychological impact. Compared with the other clusters, physicians noted that these patients were unhappy about the recurrent and restrictive nature of their disease and were more likely to be worried that their symptoms were indicative of something more serious (Table 3). Physicians often described these patients as wanting to ‘take control of their symptoms’.

### Patients with ‘inconveniencing GERD’

Patients in this cluster accounted for the largest proportion of the GERD patient population (48%; Europe, 47%; USA, 48%). Generally, these patients experienced less frequent and/or relatively mild symptoms, with lower impact, that could be adequately controlled with the therapy they were currently treated with. Symptoms were perceived by the physician to be mostly related to lifestyle, to a greater extent than in other clusters. Levels of anxiety and distress also tended to be lower in this group, who were perceived by physicians to have a low risk of future complications (Table 3).

## Discussion

This multinational survey demonstrates that physicians can generally classify GERD patients according to one of the three clusters, based on the physician-perceived impact of the disease on their physical and psychological characteristics that, in turn, influence their therapeutic needs. Given that previous research demonstrates that many GERD patients experience persistent symptoms despite prescription and/or OTC therapy (7,8), such findings could, therefore, facilitate improved patient management by allowing treatment to be tailored according to the patient's need.

Around half of the GERD population (53%) were the combined cohort of patients with ‘long-term, disrupting GERD’ and ‘recurrent, distressing GERD’. In view of the impact of the disease on their quality of life, such patients require adequate therapy to control their symptoms along with reassurance and education to address any frustrations and anxieties about their disease. However, these patients represent a challenge to physicians in terms of meeting these management goals. Patient-reported questionnaires such as the GERD Impact Scale (10), and more recently the GERD Questionnaire (GerdQ) (11) may be useful in this regard, by allowing physicians to quickly establish the impact of GERD and, in turn, the most appropriate treatment. GerdQ, for example, uses six questions regarding heartburn, regurgitation, epigastric pain, nausea, sleep disturbance and OTC medication use to identify patients with a high likelihood of GERD and those most impacted by their disease. A PPI that provides predictable and long-lasting control of GERD symptoms represents a logical choice for such patients.

With regard to cluster terminology, it is important to consider that the three clusters are not mutually exclusive and certainly for the ‘disrupting’ and ‘distressing’ categories, there is potential scope for overlap. Adopting an overall terminology to reflect these clusters was somewhat difficult. However, the notable difference is that those with ‘disrupting GERD’ had generally reached a point at which they were accepting of their symptoms. In contrast, the ‘distressed GERD’ cluster was characterised by the recurrent nature of their symptoms and a high level of distress related to restrictions on their daily lives.

Some 48% of the surveyed patient population had ‘inconveniencing GERD’, in that symptoms were occasional and/or mild, with low impact, and were perceived by physicians to be mostly related to lifestyle. These patients typically required an ‘instant solution’ to their symptoms, and OTC therapy with an antacid, or on-demand use of a prescription acid-suppressive agent, would seem appropriate to meet their needs. However, such patients should be encouraged to seek appropriate medical care should their symptoms become more troublesome (in terms of frequency and severity) and daily life is impaired.

Limitations of this study include the potential bias towards physicians willing to participate in survey research, and a possibly uneven balance of contributors (in that only 24% of participants were GI specialists; the remainder were PCPs). Also, considering the fact that the patient clusters were derived from physicians’ perceptions of their patients’ clinical and emotional needs, the observation of a large proportion of patients with ‘inconveniencing GERD’ may have arisen from the tendency of physicians to underestimate the severity and impact of GERD symptoms, as shown previously (12). This limitation may have been addressed by validation of physicians’ perceptions against patients’ perceptions and experiences of GERD, which could form the basis of further research. However, it should be recognised that the present analysis attempted to overcome this limitation by evaluating physician responses on statements such as future risk of complications, physical evidence of disease, frequency and severity of symptoms and associated disruption. Other possible limitations include the relatively low response rate (15%), but this was deemed acceptable for this type of research. There was also the possibility of ‘forced’ classification of patients into one of the five patient clusters identified as part of the preceding qualitative research. However, physicians were able to classify all of their patients according to one or the other of the five pen portraits, suggesting that the qualitative hypothesis was strong. Additionally, there may have been an over-representation of patients who visited their physician more frequently and, therefore, had a higher chance of being recalled by the physician than patients who rarely sought medical care.

## Conclusion

In summary, patients presenting to their physician with GERD can generally be classified as having ‘long-term, disrupting GERD’, ‘recurrent, distressing GERD’ or ‘inconveniencing GERD’, based on the physician-perceived impact of the disease on physical and psychological characteristics. Recognition and understanding of these population clusters may facilitate improved management of patients with GERD by allowing treatment to be tailored according to the patient's need.
